# Frequency and behavior of *Melipona* stingless bees and orchid bees (Hymenoptera: Apidae) in relation to floral characteristics of vanilla in the Yucatán region of Mexico

**DOI:** 10.1371/journal.pone.0306808

**Published:** 2024-07-24

**Authors:** José Javier G. Quezada-Euán, Roger O. Guerrero-Herrera, Raymundo M. González-Ramírez, David W. MacFarlane

**Affiliations:** 1 Departamento de Apicultura Tropical, Campus Ciencias Biológicas y Agropecuarias, Mérida-Xmatkuil, Mexico; 2 Department of Forestry, Michigan State University, East Lansing, MI, United States of America; University of Carthage, TUNISIA

## Abstract

*Vanilla planifolia* is native to the Mexican tropics. Despite its worldwide economic importance as a source of vanilla for flavoring and other uses, almost all vanilla is produced by expensive hand-pollination, and minimal documentation exists for its natural pollination and floral visitors. There is a claim that vanilla is pollinated by *Melipona* stingless bees, but vanilla is more likely pollinated by orchid bees. Natural pollination has not been tested in the Yucatán region of Mexico, where both vanilla and potential native bee pollinators are endemic. We document for the first time the flowering process, nectar production and natural pollination of *V*. *planiflora*, using bagged flower experiments in a commercial planting. We also assessed the frequency and visitation rates of stingless bees and orchid bees on flowers. Our results showed low natural pollination rates of *V*. *planifolia* (~ 5%). Only small stingless bees (*Trigona fulviventris* and *Nannotrigona perilampoides*) were seen on flowers, but no legitimate visits were witnessed. We verified that there were abundant *Euglossa* and fewer *Eulaema* male orchid bees around the vanilla plants, but neither visited the flowers. The introduction of a colony of the stingless bee *Melipona beecheii* and the application of chemical lures to attract orchid bees failed to induce floral visitations. *Melipona beecheii*, and male orchid bees of *Euglossa viridissima and E*. *dilemma* may not be natural pollinators of vanilla, due to lack of attraction to flowers. It seems that the lack of nectar in *V*. *planifolia* flowers reduces the spectrum of potential pollinators. In addition, there may be a mismatch between the attractiveness of vanilla floral fragrances to the species of orchid bees registered in the studied area. Chemical studies with controlled experiments in different regions would be important to further elucidate the potential pollinators of vanilla in southern Mexico.

## Introduction

The pantropical genus *Vanilla* (Plumier ex Miller) is probably the most widely appreciated of all orchids, thanks to the famous flavoring extract that is found in its pods [1]. The Mexican-native species, *Vanilla planifolia*, is the most commonly used commercial vanilla. Despite its worldwide economic relevance, the natural reproduction of *V*. *planifolia* and the role of potential flower pollinators has been little studied in its center of origin.

*V*. *planifolia* is a perennial, hemi-epiphytic climbing succulent vine [2]. The inflorescence develops laterally from the leaf axil, with short-lived, yellowish-green flowers lasting less than a day [2]. Some species of *Vanilla* yield fruit through spontaneous self-pollination and this form of autogamy has been reported for *V*. *planifolia* [3]. However, data on commercial cultivars generally report low fruit set, for which there is no clear explanation [4, 5]. It is generally assumed that such low productivity may be associated with insufficient numbers or absence of natural pollinators [4]. Indeed, under commercial production, vanilla flowers need to be hand-pollinated to ensure sufficient production of fruit, rendering vanilla as one of the few crops whose productivity depends entirely on hand-pollination, even in its region of origin [2]. This agricultural practice contributes substantially to vanilla’s high prices; it is the 2^nd^ most expensive agricultural product [6]. In 2018, a steady increase in global demand, with a concomitant reduction in global supply, caused what has been called the “vanilla crisis”, which promoted an unbridled increase in the price [7]. Thus, in the light of the growing demand for vanilla, understanding the limitations of its natural production becomes increasingly relevant.

The pre-Columbian people of north-central Veracruz, Mexico, are believed to be the earliest to cultivate vanilla; however, the oldest reports of vanilla refer to its use as spice for cacao-beverage by the Maya [8]. Today, production of vanilla in Mexico is concentrated around the eastern tropical regions, encompassing the states of San Luis Potosí, Hidalgo, Puebla and Veracruz [2, 9]. In the South of Mexico, Chiapas and the Yucatán Peninsula are also home to vanilla cultivars, although cultivated at a lesser extent [10]. Notably, most vanilla production in Mexico is still artisanal, in agroforestry systems [2], which can be important for preserving forests, wildlife and traditional cultural methods.

The natural pollinators of *V*. *planifolia* remain unknown. Sporadic records have witnessed euglossine and stingless bees visiting flowers of Mexican *Vanilla* [4], but characterization of the different species and their behavior remains undescribed. The Mexican tropics are home to a rich apifauna, including 47 species of Euglossini, commonly known as ‘orchid bees’ [11] and at least 46 species of Meliponini or ‘stingless bees’, of which seven are species of *Melipona* [12]. For the Yucatán Peninsula, at least 11 species of Euglossini [13] and 16 species of Meliponini [14] are reported, including the anciently cultivated *Melipona beecheii* Bennett *(Hymenoptera*: *Apidae)* which has been claimed as the natural pollinator of vanilla [15–17]. There is an extended belief that vanilla is pollinated by *Melipona* stingless bees, and because these bees are endangered and rare, the productivity of vanilla may be compromised [5, 17]. However, as vanilla is an orchid, it is possible that it may be more readily visited by euglossine bees [18]. Indeed, some species of wild vanilla are primarily adapted to pollination by euglossine bees [19]. Nevertheless, there has been no assessment of the natural frequency and visitation rates of orchid bees or *Melipona* bees to *V*. *planifolia* in the Yucatán region of Mexico, where both vanilla and these bee species are native, and no determination if these bees can be induced to pollinate vanilla.

In this study, we documented the flowering process of *V*. *planifolia* in southern Mexico. Then, using bagged-flower experiments, we assessed natural pollination rates. At the same time, we documented the natural activity, i.e. the frequency and rates of visits by orchid bees and stingless bees, to test if pollination may be affected by the availability of both bee types. Finally, we evaluated if floral visitation could be artificially induced by increasing bee numbers, either by colony introduction of *M*. *beecheii* or by applying chemical lures to attract euglossines to flowers of vanilla.

## Materials and methods

### Study design

The study was conducted in a young vanilla planting, where at least 30 plants of *V*. *planifolia* had started their first blossom. The study was conducted with permission on private land, within the boundary of the natural forest reserve of Cuxtal, near Merida in the state of Yucatán, Mexico (20°49’54.84N 89°40’33.88 W) (S1 Fig). Secondary deciduous forest is the predominant landscape in the area and the vines were grown on live tree “tutors” of *Gymnopodium floribundum*, where no insecticides have been applied. We expected that the natural forest conditions of the reserve would provide adequate background for the presence of native bees to find the vanilla plants. Meliponaries with colonies of *M*. *beecheii* are kept throughout the reserve. Apiaries with *Apis mellifera L*. *(Hymenoptera*: *Apidae)* and feral honeybee colonies are common too [20].

The experiments were conducted when the plants started flowering in the first week of April and continued until they finished in mid-May of 2023.

### Flower anthesis and reward

We documented the cycle of flower anthesis by marking three flowers on different plants. Photographs were taken every 30 min to observe the start of the opening and the closing of the flowers (S2 Fig). This part of the study was conducted starting at 1 am and finishing at 1 pm and was important to assess if flowers could be available for nocturnal visits. *V*. *planifolia* flowers consist of three sepals (one dorsal and two lateral ones), two lateral petals, and one central trumpet-shaped petal known as the lip or labellum [21]. The flower opens for only one day, with possible access to the reproductive parts inside the labellum trumpet [2]. To assess the process of anthesis and to quantify the degree of the opening of the flowers, we measured the gradual separation between the tip of the dorsal sepal and the superior margin of the labellum every hour between 1 am and 1 pm.

To assess the presence of a nectar reward for pollinators, we used 1 and 2 ul microcapillary tubes to probe liquid from the base of the tubular lip [22]. We applied this method to previously bagged floral buds (see following section). On the day of anthesis, one experimental flower was unbagged to attempt nectar collection at each of three times, 8:00 am, 10:00 am and 12:00 pm. After each attempt, the flowers were bagged again until the following time. After the final attempt to collect nectar in the field, the experimental flowers were taken to the lab where they were dissected for the presence of nectar at the base of the labellum using a stereoscope. A total of six flowers were studied on two different days. The flowers were from different plants, one flower from each of six plants.

### Pollination rates

We studied pollination rates on 10 vanilla plants. We used bagging experiments to estimate differences in natural and controlled pollination treatments in the cultivar. For this, bags made of cloth were used to isolate floral buds 24 h before anthesis (S3 Fig). Flowers were assigned to one of three different treatments: a) ‘closed’ (bagged) to estimate the rate of autonomous self-pollination, b) ‘open’ (not bagged), to estimate the rate of natural pollination, and c) artificial pollination (not bagged) to compare the effect of hand-pollination. For the first treatment, flowers were kept with their bags on during the whole period of anthesis, after which, when the flowers withered, the bags were removed. For the open pollination treatment, bags were removed at 4 am and flowers were then left open until withering. For the artificial pollination treatment, flowers were hand-pollinated using a standard procedure, at 9 am, after which they were left open. The effectiveness of pollination was measured as the success of pod formation (fruit set) one week after treatment application. We did not wait until the ripening of the pods which could take up to nine months [2]. With the data from the different treatments, we calculated the pollen limitation index (PL) as PL = (Ps − Po)/Pmax (Ps or Po) [23], For Ps we used the number of pods from hand-pollinated flowers, for Po we used the number of pods from open flowers, and for Pmax we used the larger of the two values (in our case Ps). The index ranges between 0 and 1 when limitation is highest.

### Floral visitors: Stingless bees and orchid bees

The presence of floral visitors was assessed by direct observation of experimental plants in flower. For a period of two weeks, the flowers of three different plants were watched, each by one of three trained observers. Across seven hours flowers were observed between 6 am and 1 pm when they withered. Each observer followed two flowers per plant. A distance of 1.5 m was kept between the observer and the plant, which allowed for unimpeded observations of visits to flowers and identification of the visitors. Video cameras set close to individual flowers were also used to help in documenting visitors.

To estimate the presence of male orchid bees around the planting, we tested eight chemical compounds that have been reported as attractants for different species of euglossines [18]; these are: eugenol, 1,4-dimethoxybenzene, vanillin, methyl cinnamate, cineole, β-lonone, α-pinene and carvone.

Lures of each compound were placed in the center of the cultivar. Each lure consisted of 100 μl of compound solution applied to tissue paper placed inside a sphere of metallic mesh that was replenished every hour with the same amount of solution. Each lure was placed at a height of 1.5 m above ground and were separated 5 m from each other. The frequency of male orchid bees was followed starting at 7 am and ending at 1 pm. Every hour the observer made a quick count of the number of males flying around the lure. In the case of males of *Euglossa*, we separated males of *Euglossa viridissima* Friese and *E*. *dilemma* Bembé and Eltz (Hymenoptera: Apidae) by capturing males with a net. The number and arrangement of mandibular teeth was used to separate both species [24]. The process was done on three different days with one-week intervals (S4 Fig).

### Experiments to induce floral visitation

A hive with a colony of *M*. *beecheii* was established in the center of the vanilla planting the week the blossoms started (S5 Fig). We used a strong colony with approximately 2500 workers, with a laying queen and 8 brood combs at different stages of development. The distance between the colony and the closer experimental plant was 2 m, with the more remote one at 25 m. Two weeks after the colony’s introduction, its activity was monitored by counting the number of bees exiting and entering the hive for 10 min every hour between 6 am and 12 pm. This was done for two consecutive weeks.

From the initial experiment with chemical lures, it was evident that *Euglossa* were the most abundant male orchid bees present on the planting. We tried to attract them to flowers with what we determined to be the most attractive chemical compounds, which were eugenol and 1,4-dimethoxybenzene. We used 10 μl of each compound pipetted halfway inside the labellum tube of individual flowers. The application was done at 9 am. As a control, and to confirm the presence of orchid bees at the time of the experiment, the same amount of chemical was applied to lures using a similar procedure to the one for documenting the presence of orchid bees (see Floral visitors). A total of 4 flowers treated each with one of the two compounds (two flowers per compound), and lures of each compound 5 m apart among them were followed on two different days. The observations started at 7 am the day of anthesis and the presence of males was followed counting their numbers every hour until 2 pm.

## Results

### Flower anthesis and reward

**Fig 1:**
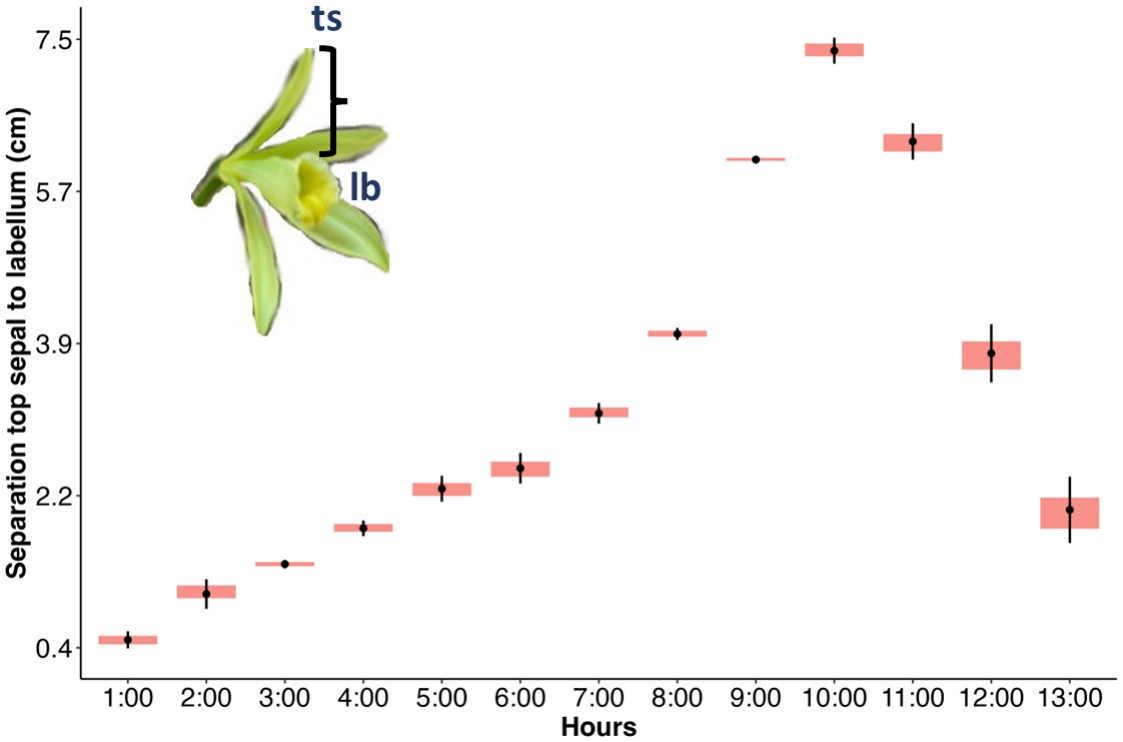
Process of vanilla flower anthesis documenting the separation of the top sepal (ts) and the superior margin of the labellum (lb) over time.

One or two flowers opened per day on the experimental plants. Flowers started opening at around 1 am with the dorsal sepal being the first to open moving upwards (Fig 1). Clearer differences in the opening of the petals started at around 3 am, and by 4 am the lateral petals started opening downwards and laterally. By 6 am the dorsal sepal, petal and lateral sepals were more separated, but the complete exposure of the labellum with the tube occurred at around 7:30 to 8 am (Fig 1 and S2 Fig). The maximum separation between the top sepal and the superior margin of the labellum (causing its maximum exposure) occurred at about 10 am (Fig 1). The labellum remained exposed until around 12 pm when the dorsal sepal with the lateral ones and the petals started closing down, restricting access to it (Fig 1 and S2 Fig). The closing of these structures progressed rapidly and by 1 pm the labellum was concealed within them (S2 Fig). This means that the flowers had a period of complete exposure of the labellum of about 4 hours, between 8 am and 12 pm (Fig 1).

Attempts to obtain nectar in the field from previously bagged flowers failed at the three times of day sampled during anthesis. When the flowers were dissected in the lab there was no evidence of nectar at the bottom end of the labellum.

### Pollination rate

To estimate pollination rates we used the results of the bagged-flower experiments. The natural pollination rate was obtained as the number of pods divided by the total number of flowers that were included in the open pollination treatment (Table 1). The value obtained for natural pollination was 4.9%, which is very low compared with the pollination rate obtained from the flowers that were hand pollinated (70%). Additionally, we did not obtain evidence of autonomous self-pollination in this cultivar, as none of the flowers that were permanently bagged produced pods. The estimated value of PL was 0.93 which corroborates the significance of natural pollination limitation in this crop.

**Table 1:** Summary of experiments to assess pollination rates of vanilla in southern Mexico. The number of flowers (fl) and the number of pods produced are presented from which the pollination rate was estimated for each treatment (%). In parentheses the number of pods produced per experimental flowers per plant and in superscript the number of flowers where bees were observed.

TREATMENT	PLANTS										fl	Pods	%
	1	2	3	4	5	6	7	8	9	10			
**Hand pollinated**	4 (3)	6 (5)^3^	6 (4)^1^	4 (3)	5 (2)^1^	4 (3)	3 (3)^2^	5 (3)	4 (2)	3 (1)^1^	44^8^	31	70.4
**Open**	5	6 ^2^	6 ^2^	4	4 ^2^	3	3 (1) ^1^	4 ^3^	3	3 (1)	41^10^	2	4.9
**Bagged**	4	6	6	3	5	3	2	4	4	2	39	0	0.0
**Total**											**124**	**33**	

### Floral visitors: Stingless bees and orchid bees

**Fig 2:**
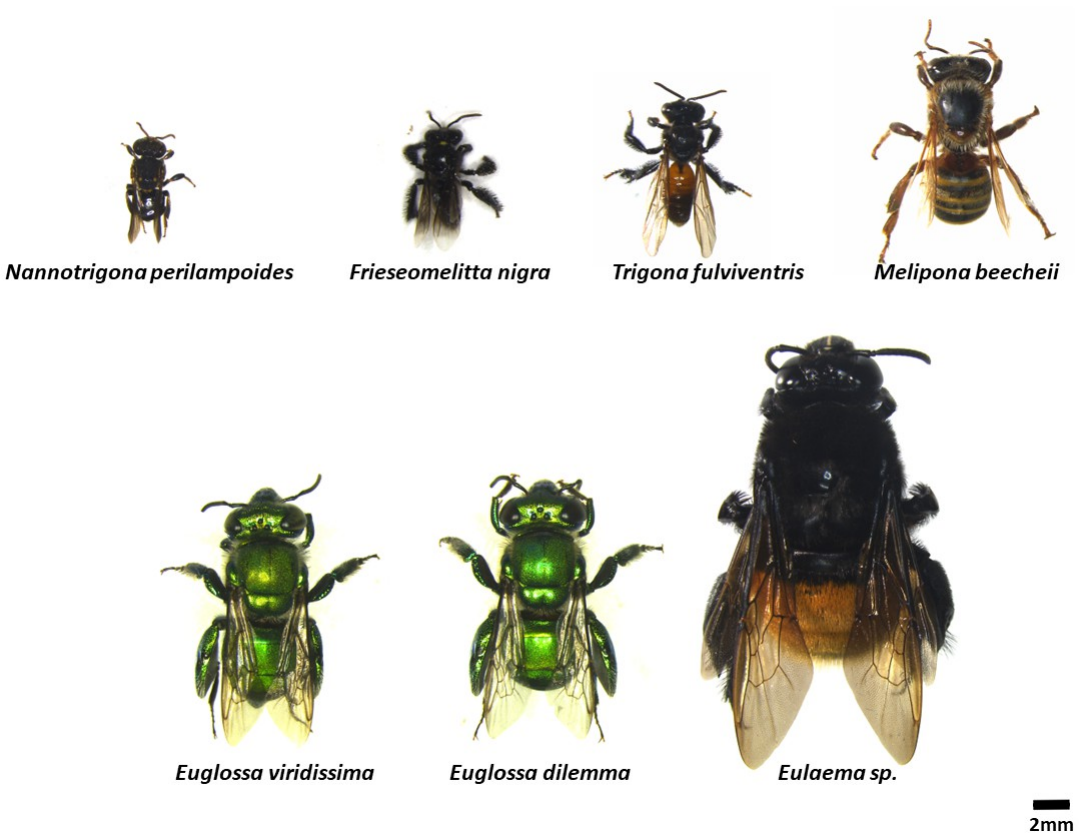
The different bee species found on the vanilla planting in the Yucatan are shown with their relative body size. Specimens on the top row are stingless bees and on the bottom row are orchid bees. A colony of M. beecheii was introduced into the planting.

A total of 76 flowers were observed for the presence of bees or other visitors for a total of 63 hours of observation time. Six bee species were witnessed, and one was introduced into the experimental vanilla planting (Fig 2). During the experiments, it was possible to see a few bees, specifically minute stingless bees of the species *Nannotrigona perilampoides* Cresson and *Trigona fulviventris* Guérin (Hymenoptera: Apidae). None of those bees were abundant, the most frequent was *T*. *fulviventris* that was recorded 17 times out of 18 bee sightings (Table 1, Fig 2). Apart from these species, no other bee species were witnessed in the vanilla planting, despite the presence of meliponaries and apiaries in the area.

The stingless bees that were present did not visit the flowers in a manner that would allow pollination. They mostly approached the flower humming and inspecting; after this they just flew away. On the few occasions when they landed on the labellum, they slowly walked on its surface, but did not enter the cone (S6 Fig). Evidently, they were not strongly attracted to the blossoms, as no recruitment of other members of the colony occurred. This is notable, considering that stingless bees are highly social and can direct their foraging force towards attractive resources by communicating back to the colony.

**Fig 3:**
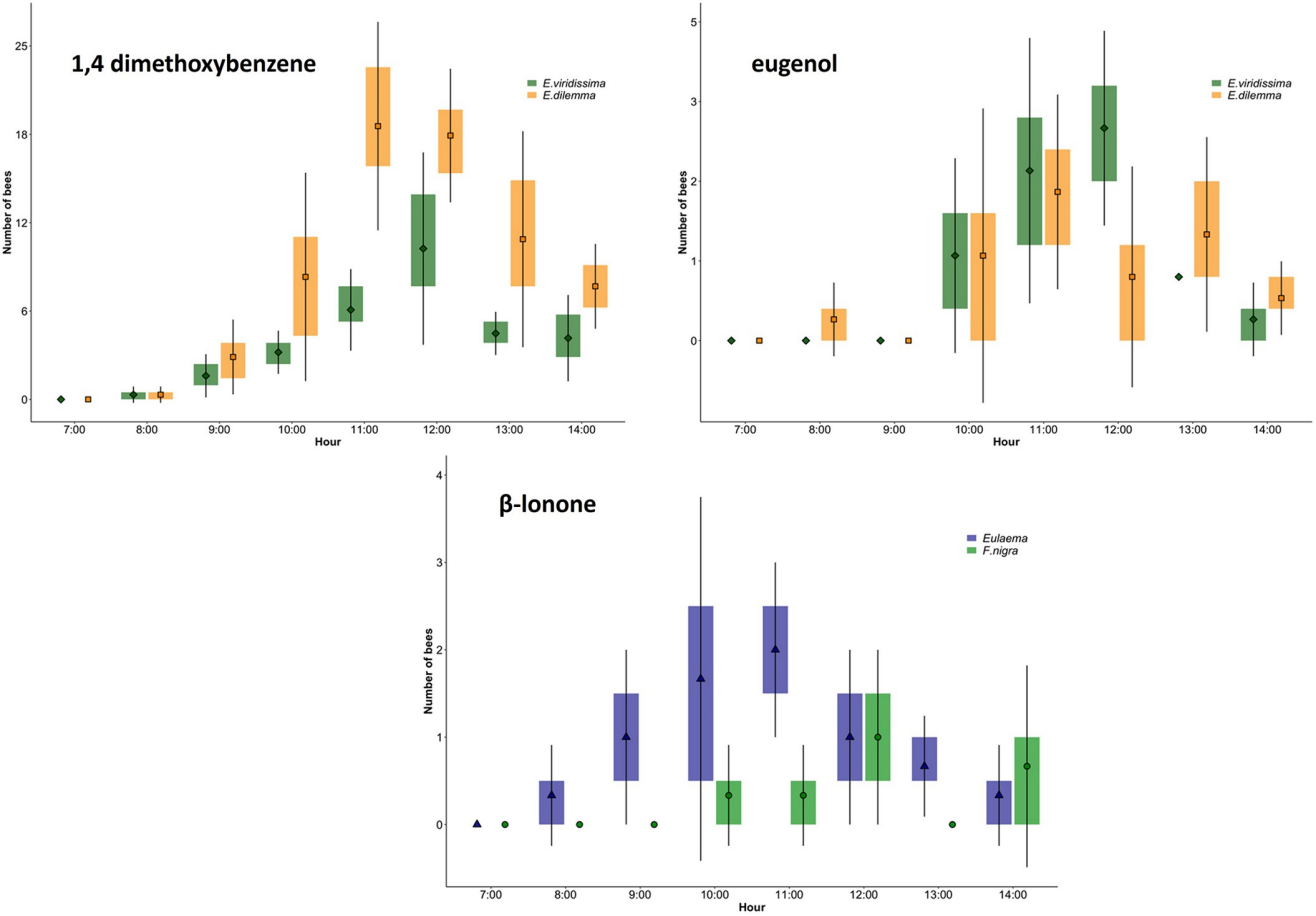
Box plots of the frequency of male orchid bees counted on lures of 1,4 dimethoxybenzene, eugenol and β-lonone placed in the vanilla plot. Squares, diamonds, circles and triangles indicate the mean values at each time.

To assess if orchid bees were present in the area, we used different chemicals. Two chemicals did not attract orchid bees, these were carvone and methyl cinnamate. Vanillin, cineole and α-pinene attracted only a few individuals. The three most attractive chemicals were in ascending order β-lonone, eugenol and 1,4 dimethoxybenzene (Fig 3). The latter was by far the most attractive compound of the eight we used. However, it mostly attracted male orchid bees of the species *E*. *viridissima* and *E*. *dilemma* (Fig 3). Eugenol also attracted male bees of both species, but in lesser numbers (Fig 3). Male orchid bees of the genus *Eulaema* were attracted only by β-lonone, but were rare, with only between 5 to 10 bees sighted per day (Fig 3). This chemical also attracted a few stingless bees of the species *Frieseomelitta nigra* Cresson (Hymenoptera: Apidae) (Fig 3). The most abundant species were *E*. *viridissima* and *E*. *dilemma*, with up to 17 males counted on the 1,4 dimethoxybenzene lure at the peak of activity (Fig 3).

### Experiments to induce floral visitation

**Fig 4:**
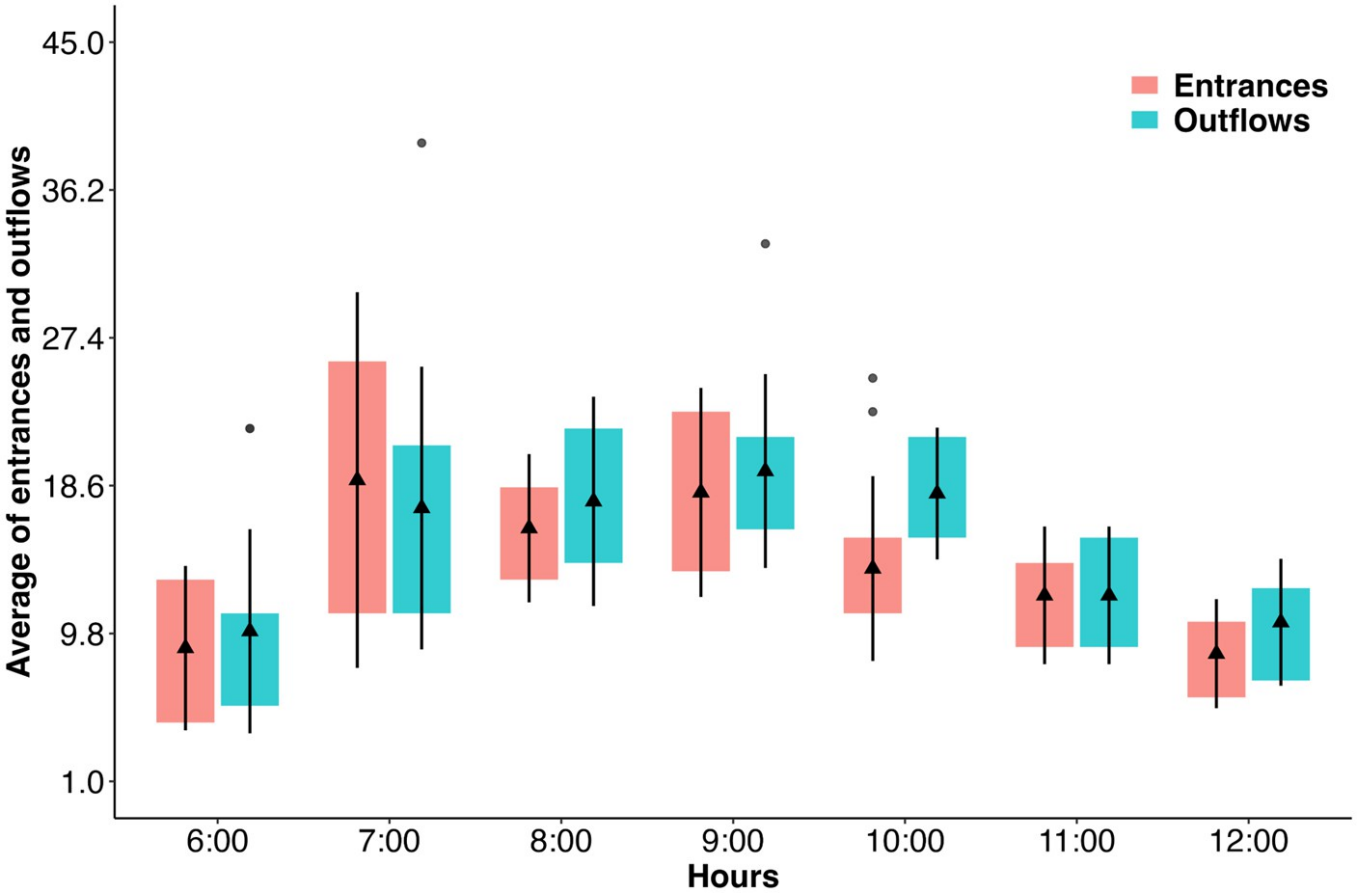
Box plot of the daily activity of *M*. *beecheii* workers measured by the number of bees entering and exiting a colony experimentally placed in the vanilla plot. Triangles indicate mean values.

The activity of the colony of *M*. *beecheii* introduced into the plot was followed for 15 consecutive days. The results show that the colony had intense foraging activity, judging by the number of bees entering and exiting the hive (Fig 4). This reached a maximum between 7 am and 9 am (Fig 4). Despite the intense foraging, no *M*. *beecheii* was observed approaching open vanilla flowers.

**Fig 5:**
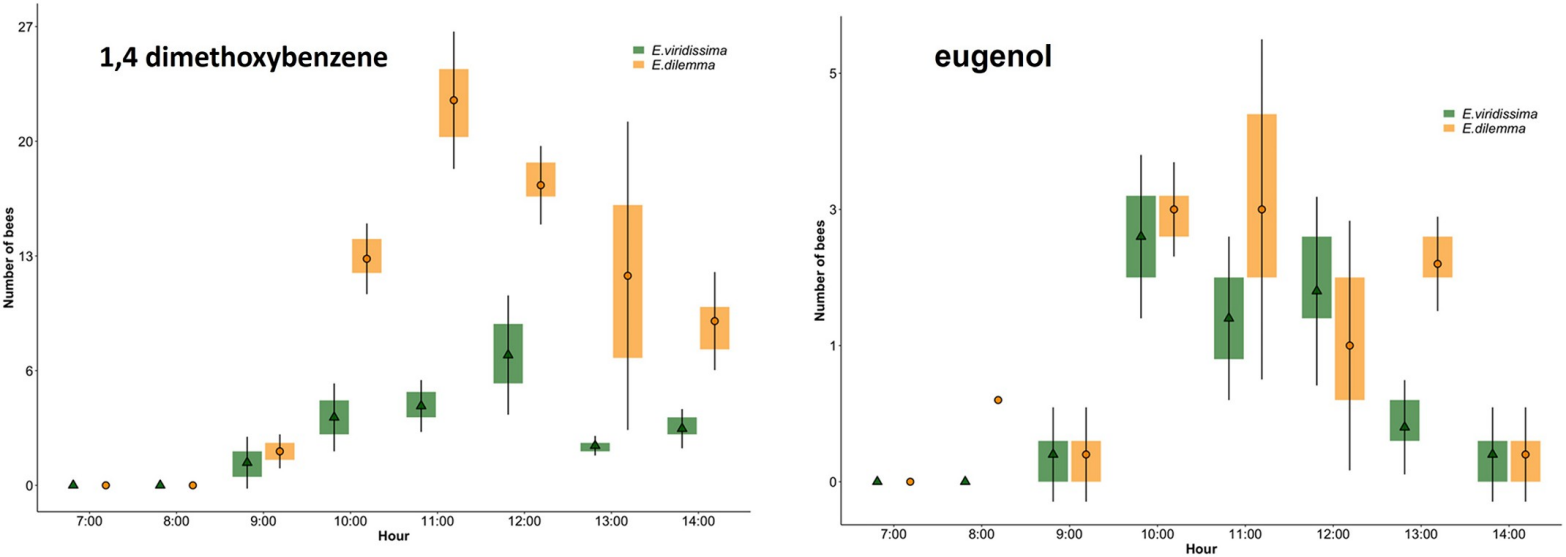
Box plots of the daily activity of *Euglossa* males as measured by the number of bees on 1,4 dimethoxybenzene and eugenol chemical lures placed in the vanilla plot during the application of the same chemical attractants to flowers. Circles and triangles indicate the mean values at each time.

The activity of male orchid bees was followed after the application of 1,4 dimethoxybenzene and eugenol to the labellum of experimental flowers. Although the frequency of males within the plot was high at the time of experiments (Fig 5), they did not go inside the flowers in which the chemicals were applied. A total of seven *Euglossa* males flew close the treated flowers with 1,4 dimethoxybenzene but did not land on them. On two other occasions males landed on the labellum treated with the same compound, but after quickly inspecting the flower, they flew away and did not enter the tube. Only two males flew close to the eugenol treated flowers and no landings on the labellum were observed.

## Discussion

Vanilla production depends on an immense workforce to carry out hand pollination of large numbers of flowers, which are each only open for a day. Therefore, studies aimed at identification of natural pollinators are of major financial relevance to vanilla’s production cost. In our work, we recorded the natural presence of potential pollinators and conducted controlled experiments to estimate natural pollination rate in the Yucatán, in southern Mexico, an area where vanilla is endemic, and its native pollinator(s) could be present. We also investigated the anthesis and floral rewards of vanilla to help understand its relationship with possible pollinators. At the same time, we quantified the presence of native stingless bees and orchid bees, the absence of which could be one basic factor limiting pollination [25], after which we artificially introduced and tried to lure these bees into the crop to counterbalance a possible numerical deficit.

We found no production of pods in permanently-bagged flowers, which corroborates the need for an external agent to promote pollination. The natural pollination rate of vanilla flowers in our study area the Yucatán was low, matching previous findings in other parts of Mexico [2, 5]. Accordingly, pollen limitation is high in this crop. Unfortunately, we were unable to observe the specific pollinator species involved. It was noteworthy that orchid bees were not scarce in the vanilla planting area. *Euglossa viridissima* orchid bees, which have been considered one of the most likely pollinators of vanilla flowers [2–4, 26] were highly abundant in the planting area, together with its sister species *E*. *dilemma*. *Eulaem*a males were also found, albeit in lesser numbers. Nonetheless, none of these euglossines were seen visiting the flowers, suggesting that male orchid bees of *E*. *viridiisma* and *E*. *dilemma* may not be natural pollinators of vanilla.

Effective pollinators usually match their activity with floral anthesis. Indeed, the performance of a visitor depends on the temporal matching between its activity and temporal patterns of reproductive maturation of the flowers [27]. We found that vanilla in the Yucatán blossoms between the end of March and mid-May, like other regions of the Northern hemisphere where it is cultivated [2]. By analyzing the process of floral anthesis, we found that the complete exposure of the labellum, with possible maximum receptivity occurring between 8 am and 12 am, making nocturnal pollination unlikely. Experiments have shown that maximum fruit set occurs after hand pollination at about 8 am and gradually decreases, presumably linked with the degree of receptivity of the stigma [28]. This information could be useful to evaluate potential vanilla pollinators.

In the case of *Euglossa* males, their maximum peak activity was between 11 and 1 pm, when vanilla flowers were at the end of the period of maximum labellum exposure and presumably less receptive [28]. Significantly, fewer individuals were recorded between 8 and 10 am when the labellum is most exposed. It was noteworthy that *Eulaema* bees were more frequently recorded around 10 am, coinciding with the time of maximum labellum exposure, but their numbers were low.

Another commonly believed pollinator of vanilla is the Mayan stingless bee *M*. *beecheii* [15, 29]. Although colonies of this species were reported in the study area, we observed no natural visits by this bee to the vanilla flowers. With a lack of natural visitation of vanilla flowers by *M*. *beecheii* bees, we tried to induce visitation by introducing a colony of them. The patterns of foraging of the *M*. *beecheii* colony placed in the cultivar indicated maximum activity between 7 and 10 am, which coincided with the period of labellum exposure in the vanilla flowers. Nonetheless, the bees were not attracted to the flowers, despite having a colony with strong foraging force, as seen from the results of colony activity, placed only a few meters away from them.

For species of vanilla included in the *planifolia* group, the attraction to potential pollinators seems to rely on a combination of perfume release, which should attract male euglossine bees, and nectar seeking, since insects must enter the tube formed by the column and labellum when searching for nectar [19, 30]. However, applying the most attractive chemical compounds for euglossine bees caused only a few males to approach the vanilla flowers, but also failed to induce pollination.

From the evidence we gathered, *V*. *planifolia* does not seem to produce nectar. The collection of nectar using microcapillary tubes and the dissection of flowers failed to detect nectar inside them. This finding agrees with recent evidence, indicating that although nectaries and their related secretory tissue are present in the flowers of *V*. *planifolia*, these do not seem to effectively secrete nectar [30]. Thus, *V*. *planifolia* seems to conform with the orchid type that attract pollinators by food deception. Reward-producing flowers characterize South and Central American species of vanilla, while deceptive pollination is prominent in the clade including North American taxa in which pollinators are attracted by fragrances [31]. It has been consistently found that nectarless species of *Vanilla* set less fruit compared with nectariferous species [32]. This may be due because in the case of nectariferous species, pollinators visit flowers more frequently and spend more time on them [33]. Our results indicate that the number of potential pollinators may also be reduced due to the lack of nectar in the flowers of *V*. *planifolia*.

One explanation for the absence of nectar in *V*. *planifolia* is that the production of floral rewards can be energetically expensive, and that the energy expenditure associated with reward production can be more usefully allocated to other functions [30, 34]. The lack of nectar secretion may explain why stingless bees (or generalist honeybees) were not strongly attracted to the vanilla flowers. Studies have shown that foraging *Melipona* bees exhibit preference for high sugar rewards (between 27 to 60%) in floral nectar [35]. In particular, *M*. *beecheii* prefers nectar with high sugar concentration, above 45% [36]. Colonies of highly eusocial bees rely on finding abundant and rich resources as they are necessary for maintaining hive dynamics and metabolism [37]. Eusocial stingless bee and honeybee colonies can rapidly recruit individuals for the exploitation of profitable nectar sources as soon as they become available. Thus, richer nectar should promote more extensive floral visitation by highly social bees [35]. This mechanism can explain why vanilla was not attractive to *M*. *beecheii* nor *A*. *mellifera* in this study.

Interestingly, we found explorers of minute stingless bees *T*. *fulviventris* and *N*. *perilampoides* around the flowers. Although *T*. *fulviventris* is a species that usually collects nectars with low sugar concentrations [36], its presence in the vanilla plots was not related to nectar collection. Perhaps these bees could be attracted to the natural scents of vanilla flowers. Minute stingless bees have only rarely been observed actively collecting vainillin from chemical lures [38], but their use remains to be discovered.

Our results with the chemical lures on orchid bees suggest that pollination success in vanilla with a food deceptive system might be related to the fragrance attractiveness of flowers. We found that the two most abundant species of orchid bee were *E*. *viridissima* and *E*. *dilemma*. These species were strongly attracted to the lures of 1,4 dimethoxybenzene and eugenol. On the other hand, the less frequent *Eulaema* was only seen on β-lonone lures. Partial information is available [39] stating the presence of the chemicals 1-2-dimethyl-cyclopentane, ethyl acetate, 1-8-cineol and ocimene-trans in *V*. *planifolia* flowers [5]. It is also believed that *E*. *viridissima* is trongly attracted to 1–8 cineol [4]. However, in our experiments we found that cineole was not attractive to either *E*. *viridissima*, or its sister species *E*. *dilemma*. Both species were more attracted to 1,4 dimethoxybenzene and eugenol. As none of these chemicals are currently reported in vanilla flower scents, the low attraction of vanilla may be due to a mismatch between the resources that *Euglossa* bees search for and those offered by the vanilla flowers.

In summary, *V*. *planifolia* seems pollinated through food deception as the flowers offer no reward to potential pollinators [31]. This characteristic may reduce the spectrum of potential visitors compared to species that produce nectar. In this regard, *Melipona* and other stingless bees would not find the incentive of profitable nectar rewards needed by their colonies to visit vanilla flowers, resulting in low attraction and thus, rendering them as non-important pollinators. Our results do not agree with the general notion of absence of bees as one cause of low fruiting in vanilla. Rather, there may be a mismatch between the presence of certain species of orchid bee and the chemical attraction from the vanilla flowers. Further experiments are needed to analyze the effect of different chemicals on native orchid bees and the frequency of visits and pollination of vanilla in different geographic regions. Controlled experiments introducing stingless bees other than *M*. *beecheii* [40], and orchid bee nests of different species in boxes already designed in Yucatán [41, 42] should be key to pursue such investigation.

## Supporting information

S1 Fig Vanilla planting where the experiments were conducted. (PNG)

S2 Fig Process of vanilla flower anthesis through photographs at different times across the day. (PNG)

S3 Fig Bagged vanilla floral buds previous to anthesis and bagged-flower experiments. (PNG)

S4 Fig Placing chemical lures in metal mesh spheres, to register the presence of orchid bees. *Eulaema* and *Euglossa* males around the lures and field identification of *Euglossa* species.
(PNG)

S5 Fig Experimental hive of *M*. *beecheii* in the planting and counts of activity. (PNG)

S6 Fig *T*. *fulviventris* on the labellum of vanilla flowers. The bees were not seen entering the cone.
(PNG)

S1 File Inclusivity in global research. (DOCX)
