# Supplementary figures and images for: Frequency and behavior of Melipona stingless bees and orchid bees (Hymenoptera: Apidae) in relation to floral characteristics of vanilla in the Yucatán region of Mexico
Source: PLoS One. 2024 Jul 24;19(7):e0306808. doi: 10.1371/journal.pone.0306808 (PMC11268612; doi:10.1371/journal.pone.0306808)

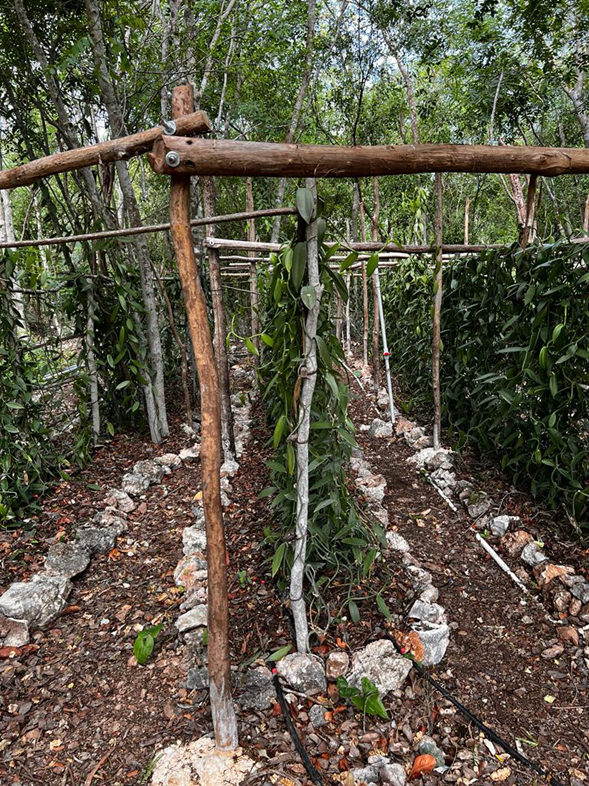

Supplement: S1 Fig — (PNG) [file pone.0306808.s001.png]

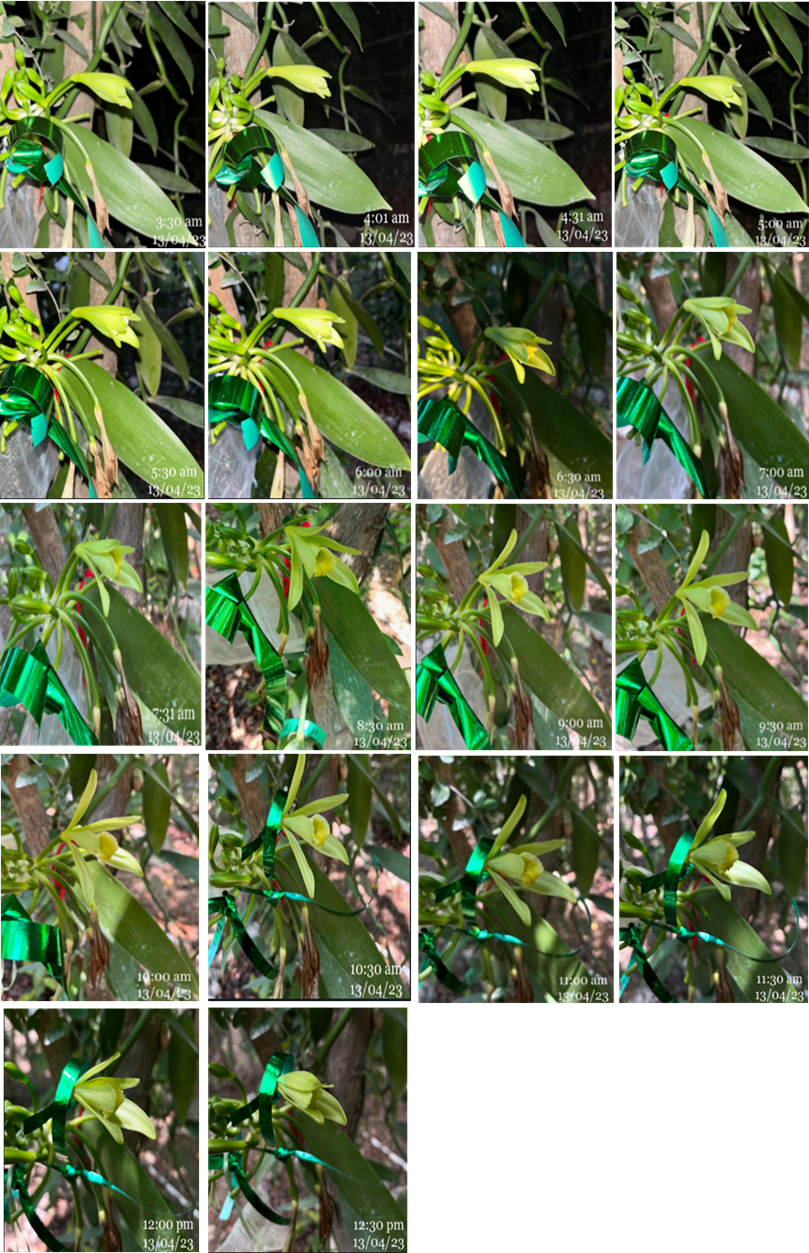

Supplement: S2 Fig — (PNG) [file pone.0306808.s002.png]

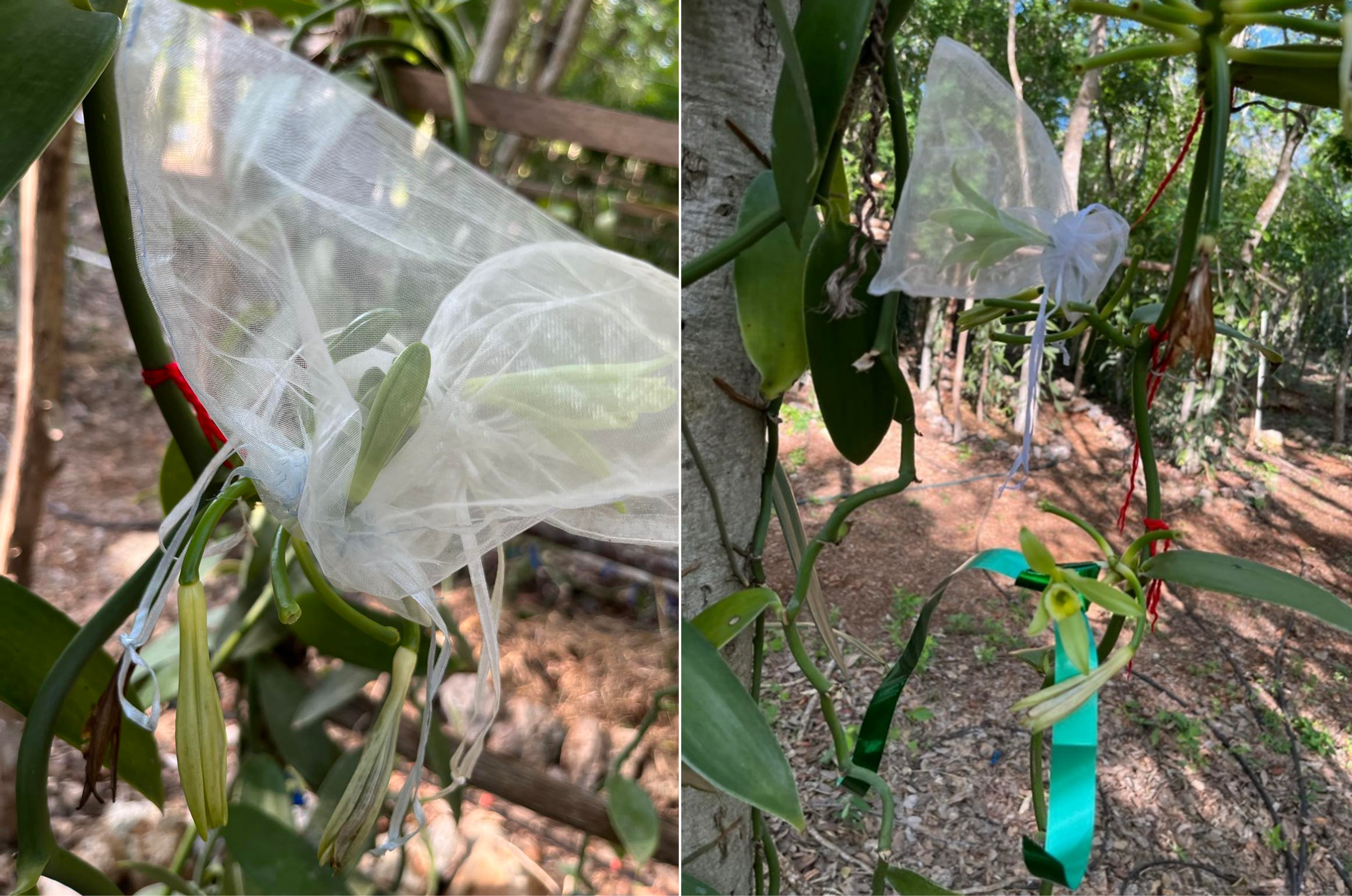

Supplement: S3 Fig — (PNG) [file pone.0306808.s003.png]

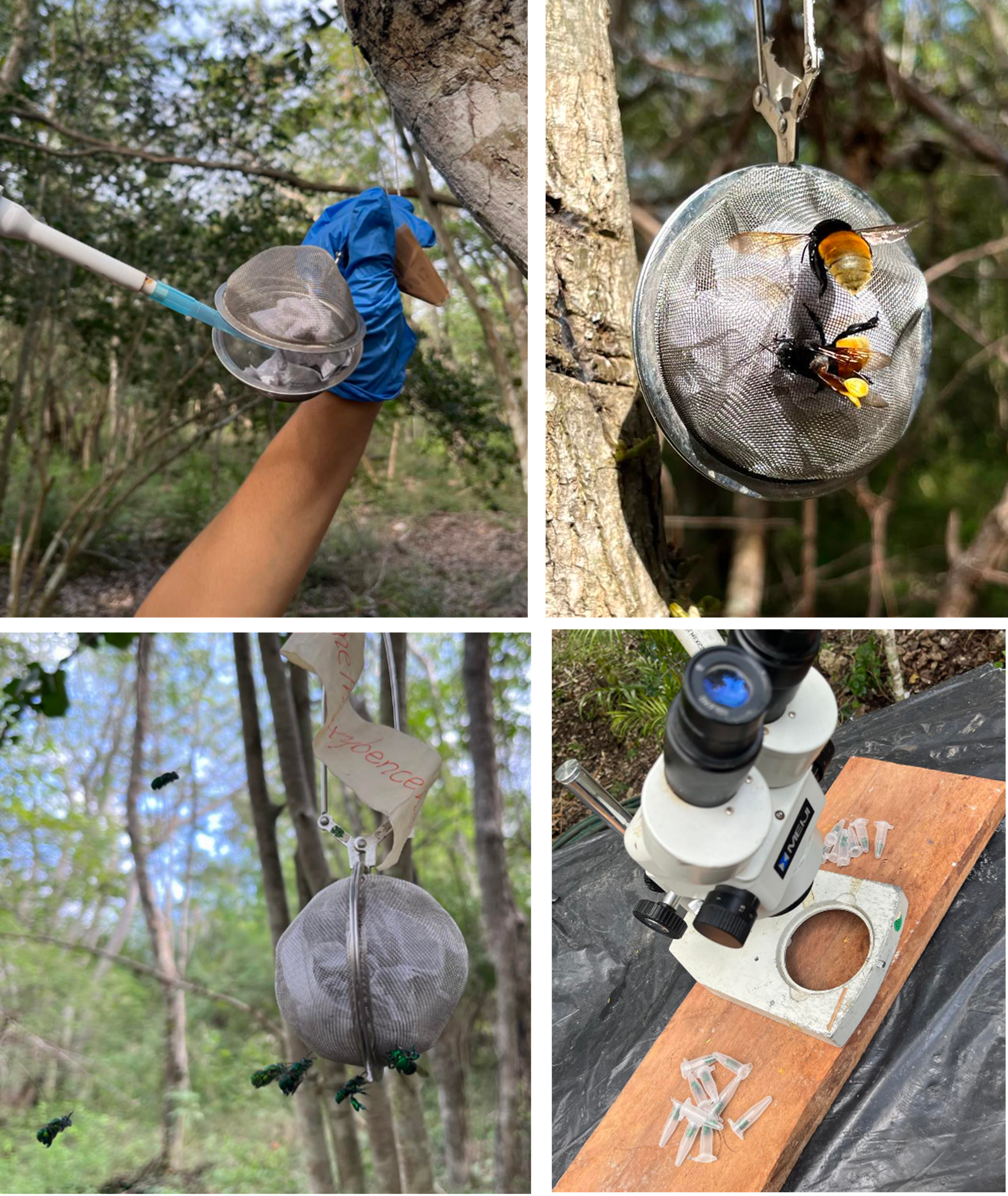

Supplement: S4 Fig — Eulaema and Euglossa males around the lures and field identification of Euglossa species. (PNG) [file pone.0306808.s004.png]

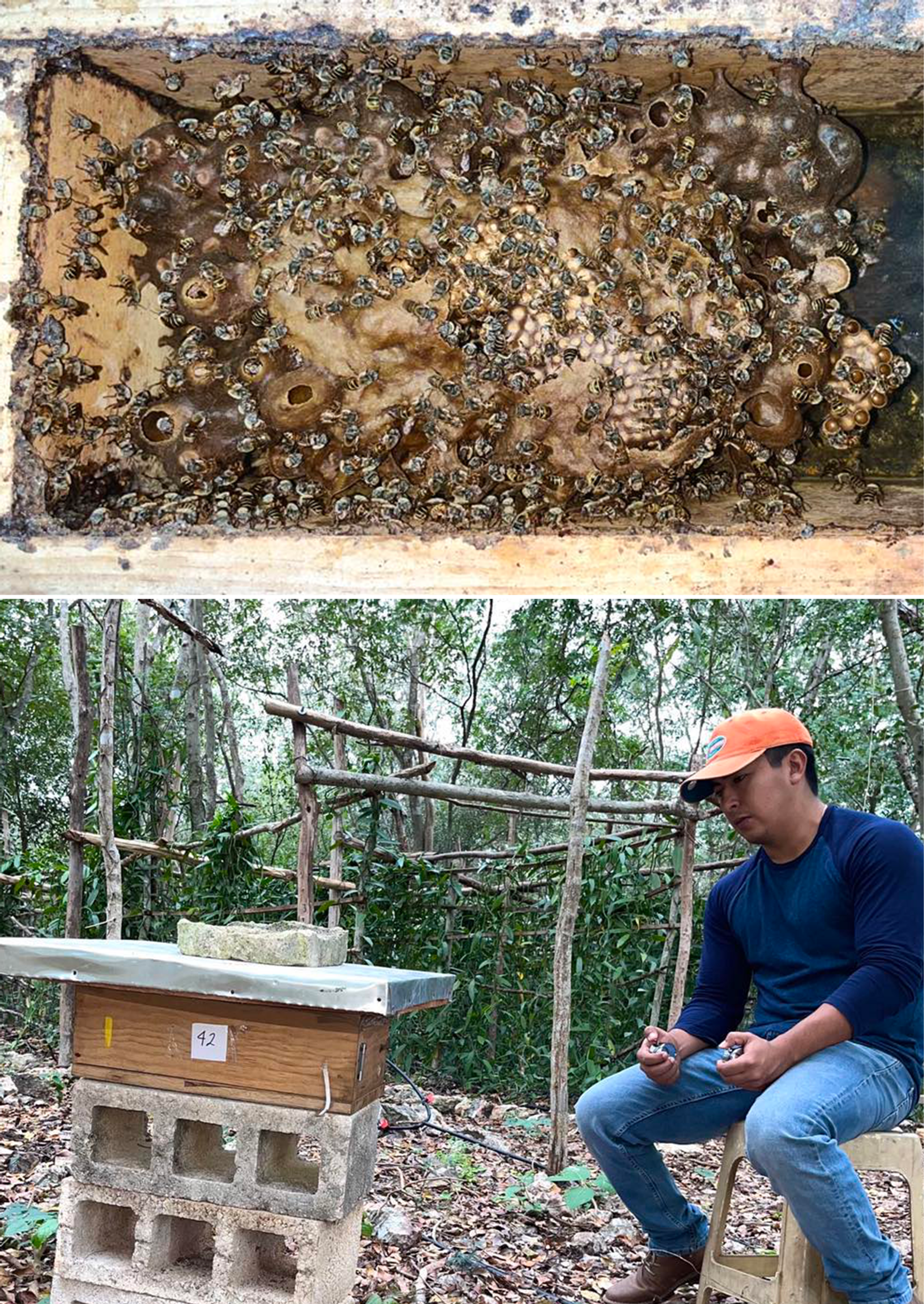

Supplement: S5 Fig — (PNG) [file pone.0306808.s005.png]

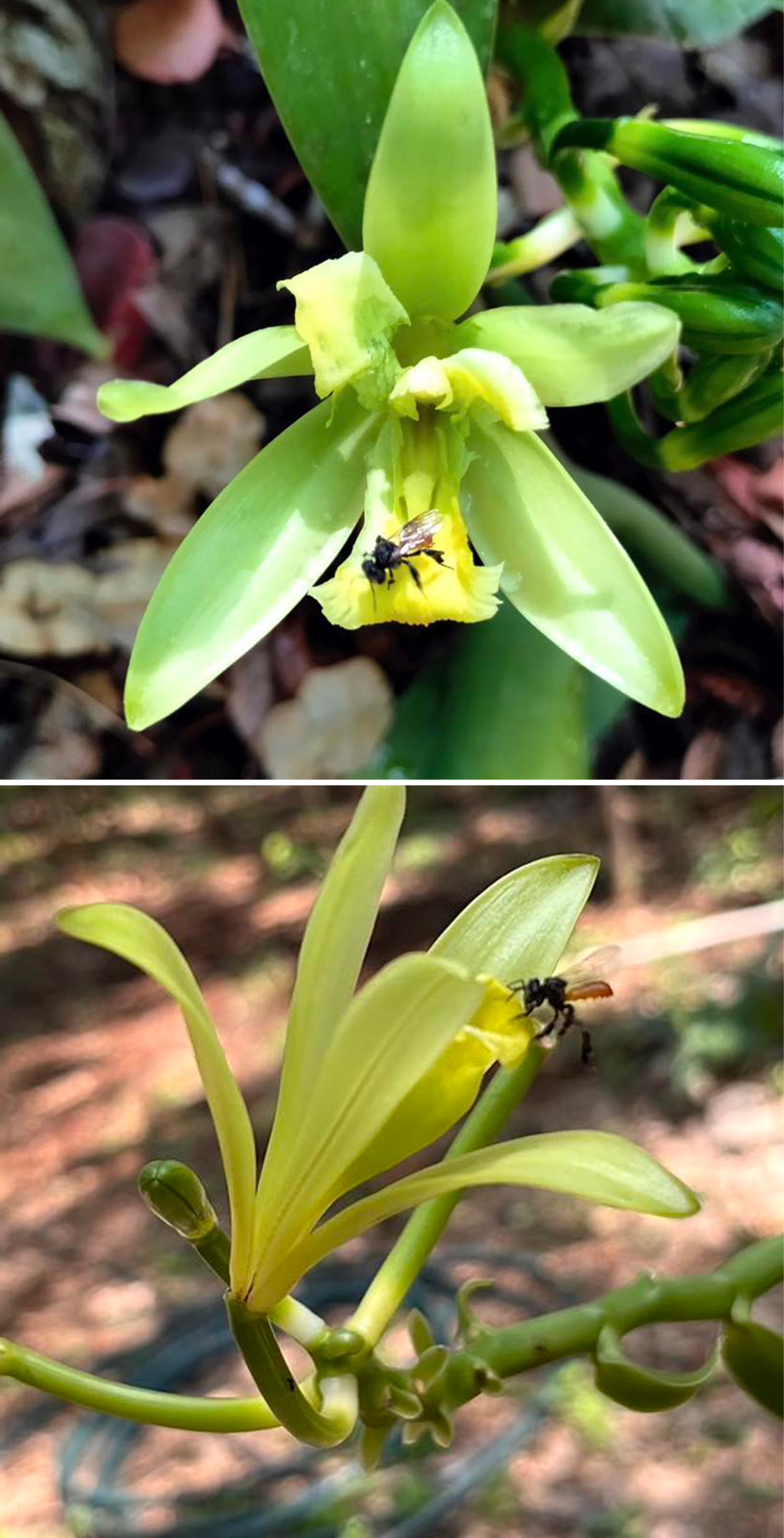

Supplement: S6 Fig — The bees were not seen entering the cone. (PNG) [file pone.0306808.s006.png]
